# Cytotoxicity of NiO and Ni(OH)_2_ Nanoparticles Is Mediated by Oxidative Stress-Induced Cell Death and Suppression of Cell Proliferation

**DOI:** 10.3390/ijms21072355

**Published:** 2020-03-28

**Authors:** Melissa H. Cambre, Natalie J. Holl, Bolin Wang, Lucas Harper, Han-Jung Lee, Charles C. Chusuei, Fang Y.S. Hou, Ethan T. Williams, Jerry D. Argo, Raja R. Pandey, Yue-Wern Huang

**Affiliations:** 1 Department of Biological Sciences, Missouri University of Science and Technology, Rolla, MO 65409-1120, USA; mhcxv8@mst.edu (M.H.C.); njhcm8@mst.edu (N.J.H.); bwnt5@mst.edu (B.W.); lh938@mst.edu (L.H.); 2 Department of Natural Resources and Environmental Studies, National Dong Hwa University, Hualien 97401, Taiwan; hjlee@gms.ndhu.edu.tw; 3 Department of Chemistry, Middle Tennessee State University, Murfreesboro, TN 37132, USA; charles.chusuei@mtsu.edu (C.C.C.); etw2r@mtmail.mtsu.edu (E.T.W.); jda6c@mtmail.mtsu.edu (J.D.A.); rajaram.pandey@mtsu.edu (R.R.P.); 4 Department of Biomedical Sciences, University of Wisconsin-Milwaukee, Milwaukee, WI 53211, USA; fyshou@gmail.com

**Keywords:** nanoparticles, viability, cell proliferation, physicochemical properties, oxidative stress, caspase-3, mitochondrial membrane potential, cell cycle, apoptosis

## Abstract

The use of nanomaterial-based products continues to grow with advancing technology. Understanding the potential toxicity of nanoparticles (NPs) is important to ensure that products containing them do not impose harmful effects to human or environmental health. In this study, we evaluated the comparative cytotoxicity between nickel oxide (NiO) and nickel hydroxide (Ni(OH)_2_) in human bronchoalveolar carcinoma (A549) and human hepatocellular carcinoma (HepG2) cell lines. Cellular viability studies revealed cell line-specific cytotoxicity in which nickel NPs were toxic to A549 cells but relatively nontoxic to HepG2 cells. Time-, concentration-, and particle-specific cytotoxicity was observed in A549 cells. NP-induced oxidative stress triggered dissipation of mitochondrial membrane potential and induction of caspase-3 enzyme activity. The subsequent apoptotic events led to reduction in cell number. In addition to cell death, suppression of cell proliferation played an essential role in regulating cell number. Collectively, the observed cell viability is a function of cell death and suppression of proliferation. Physical and chemical properties of NPs such as total surface area and metal dissolution are in agreement with the observed differential cytotoxicity. Understanding the properties of NPs is essential in informing the design of safer materials.

## 1. Introduction

Nanomaterials have become increasingly popular in the production of a wide range of products and processes including, but not limited to, cosmetics [1], pharmaceuticals [2], medical research [3], semiconductor fabrication [4], food [5], and electronic manufacturing [6]. The global market for nanomaterial-based products is estimated to reach $55 billion by 2022, with about a 20% compound annual growth rate [7]. Increased use of nanoparticles (NPs) heightens the potential for human exposure, especially from airborne particles or the consumption of products containing them. Workers in various industries are at higher risk of exposure to NPs used in manufacturing via inhalation [8]. While some NPs are relatively harmless, others produce moderate to severe toxic effects. In vitro studies have demonstrated that NPs are cellularly internalized where they can cause injuries [9,10,11,12,13]. This is observed as increased reactive oxidative stress, mitochondrial dysfunction, severe DNA damage, cell cycle arrest, induction of apoptosis, and increased necrosis [14].

Toxicity depends on the physicochemical properties of NPs [15]. For instance, the crystal structure and morphology of NPs affect cytotoxicity. The amorphous form of TiO_2_ generated more reactive oxygen species (ROS) than the anatase and rutile forms [16]. Rod-shaped CeO_2_ produced toxic responses in RAW 264.7 cells while octahedron and cubic CeO_2_ elicited little change [17]. Surface charge may also influence toxicity, with positively charged ZnO producing a higher degree of toxicity than negatively charged particles in A549 cells [18]. Three iron NPs (Fe_3_O_4_, oleic acid-coated Fe_3_O_4_, and carbon-coated Fe) with different positive charges also produced toxic responses in BEL-7402 cells, with higher positive charge correlating with worse toxic responses [19]. Dissolution rate, relative available binding sites on particle surfaces, and particle surface charge of various transition metal oxides correlated with toxicity in A549 cells [20]. It is important to note that the toxicity mechanisms of NPs are not always, but can be, cell line-dependent [9,21]. For instance, NiO NPs arrested BEAS-2B cells in the G_0_/G_1_ phase of cell cycle while arrest of A549 cells occurred in the G_2_/M phase [21]. Furthermore, NiO NPs induced a higher rate of apoptosis in BEAS-2B cells than in A549 cells [21]. ZnO exposure also induced cell cycle alterations in A549 cells but not in BEAS-2B cells [9]. Collectively, morphology, surface charge, dissolution rate, and relative surface binding sites influence NP toxicity.

NiO NPs are used in coloring agents for enamels, nanowires, automotive rear-view mirrors, and more products [22]. Ni(OH)_2_ NPs are used in rechargeable battery electrodes, nickel cadmium batteries, and nickel metal hydride batteries [23]. Nickel can be released to the environment via various anthropogenic processes. It exists in various oxidation states, though Ni(II), nickel in the +2 valence state, is its prevalent form [24]. The environmental levels of Ni-associated compounds and their effective toxic concentrations are influenced by nickel’s oxidation state, agglomeration, and media (i.e., water, soil, foods, anaerobic, aerobic), as well as by interactions with other organic and inorganic matrixes. Although the environmental concentrations of nano-sized nickel are still unknown, their existence in the environment may impose risk to human health. The mechanism of toxicity is an essential element of and forms the base of risk assessment. Toxic responses upon exposure to NiO and Ni(OH)_2_ NPs have been characterized to a limited extent in in vivo and in vitro settings. Treatment with NiO or Ni(OH)_2_ NPs induced inflammation in the lungs of rodents [25,26]. NiO induced ROS and lipid peroxidation in A549 cells [27]. Oxidative stress, apoptosis, and reduced viability in the breast cancer cell line MCF-7 and the human airway epithelial cell line HEp-2 were also caused by NiO NP exposure [28]. Particulate and soluble nickel compound treatments (including NiO and Ni(OH)_2_) led to a varying degree of toxicity in modified Chinese hamster ovary CHO-K1 (AS52) cells [29]. However, Ni(OH)_2_ cytotoxicity has not been studied in human cell lines. Adverse responses to nickel NPs observed in animals and cells indicate human health could be threatened by exposure.

To date, there are no studies comparing the difference in cellular toxicity and toxicological mechanism upon exposure of HepG2 (a human hepatocellular carcinoma cell line) and A549 (a human bronchoalveolar carcinoma cell line) to NiO and Ni(OH)_2_ NPs. Further, there have been no studies on the role of suppression of cell proliferation induced by these NPs on overall toxicity. Our preliminary data suggest that Ni(OH)_2_ NPs decrease viability more significantly than NiO NPs in A549 cells. We thus hypothesized that (1) differential cytotoxicity of NiO and Ni(OH)_2_ NPs is cell line-, particle-, time-, and dose-dependent, (2) cytotoxicity is mediated by oxidative stress and subsequent cellular events including modulation of mitochondrial membrane potential and caspase-3 enzyme activity, and (3) exposure to NiO and Ni(OH)_2_ NPs alters cell cycle and suppresses cell proliferation. Our specific aims were to (1) demonstrate that cytotoxicity is cell line-, particle-, time-, and dose-dependent, (2) measure the differences in various biochemical responses upon NiO or Ni(OH)_2_ exposure, and (3) investigate whether cell viability is a function of cell killing and inhibition of cell proliferation. To achieve our goals, we measured cell viability in HepG2 and A549 cells upon NiO or Ni(OH)_2_ exposure. We then delineated the toxicological mechanism of action in the context of the physical and chemical properties of NPs and oxidative stress-mediated cellular injuries, including changes in mitochondrial membrane potential, caspase-3 activity, apoptosis, cell proliferation, and cell cycle, in A549 cells.

## 2. Results

### 2.1. Physiochemical Properties of NiO and Ni(OH)_2_

**Figure 1:**
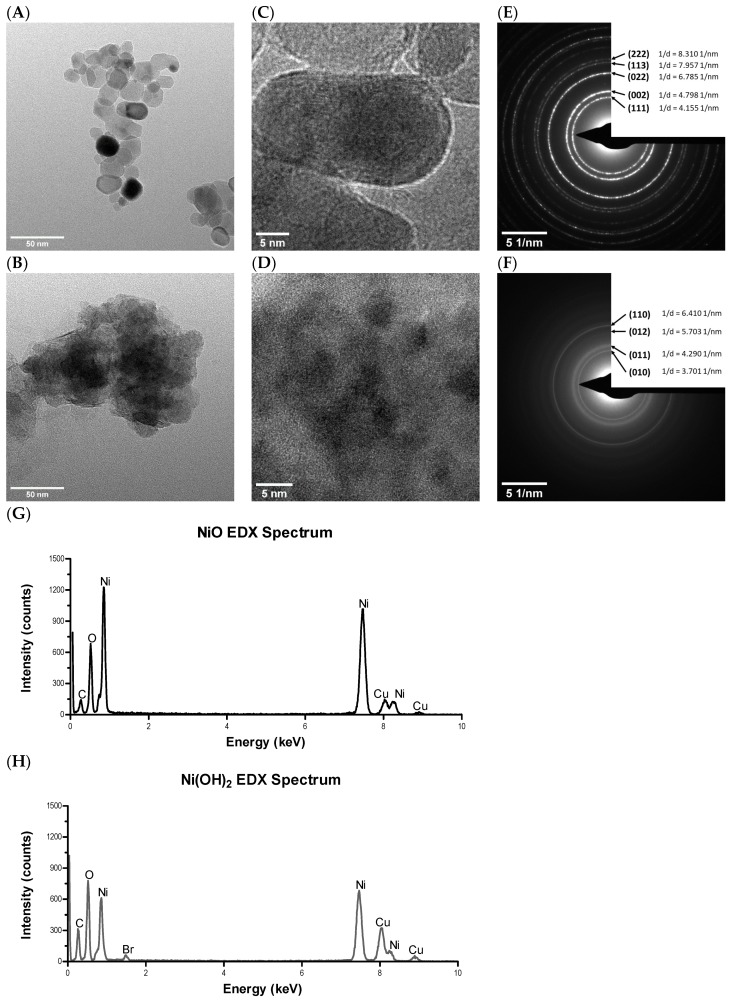
Morphology of (**A**) NiO and (**B**) Ni(OH)_2_ nanoparticles (NPs) from TEM bright field images. The lattice fringes of (**C**) NiO and (**D**) Ni(OH)_2_ polycrystals are visible from high resolution transition electron microscopy (HRTEM) images. Selected area electron diffraction (SAED) patterns of (**E**) NiO and (**F**) Ni(OH)_2_ are shown with (hkl) and 1/distance (1/nm) values for prominent rings indicated. The energy disperse X-ray spectroscopy (EDX) spectra of (**G**) NiO and (**H**) Ni(OH)_2_ show that both particles are composed of Ni and O, with C and Cu peaks being attributed to the TEM grid material.

The physiochemical properties of NiO and Ni(OH)_2_ were analyzed to determine differences between the two NPs which may contribute to distinct cellular responses (Table 1). Transition electron microscope (TEM) analysis revealed the apparent morphology and crystalline structure of the NPs (Figure 1A–F). NiO was in the form of aggregated nanograins, while Ni(OH)_2_ was a flakey aggregate. Selected area electron diffraction (SAED) patterns (Figure 1E,F) confirmed that NiO NPs have a cubic crystal symmetry with a Fm$\bar{3}$m space group, and Ni(OH)_2_ NPs have a trigonal crystal symmetry with a P$\bar{3}$m1 space group, consistent with X-ray diffraction (XRD) data (Figure S1). Both NPs contained Ni and O peaks in energy disperse X-ray spectroscopy (EDX) analysis, as expected (Figure 1G,H). Ni(OH)_2_ contained a small peak that correlated with Br. However, this contamination was estimated to be 1.01% by INCA software (ETAS, Stuttgart, Germany), excluding mass contributed by H, and was thus determined to be insignificant. Peaks corresponding to C and Cu are due to the TEM sample grid composition.

**Figure 2:**
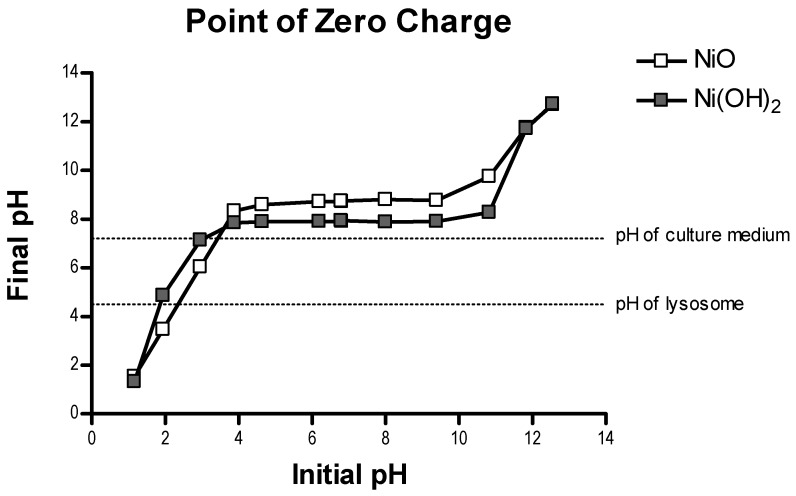
Point of zero charge (PZC) analysis of NiO and Ni(OH)_2_. Results indicate that the PZCs of NiO and Ni(OH)_2_ are 8.7 pH and 7.9 pH.

**Figure 3:**
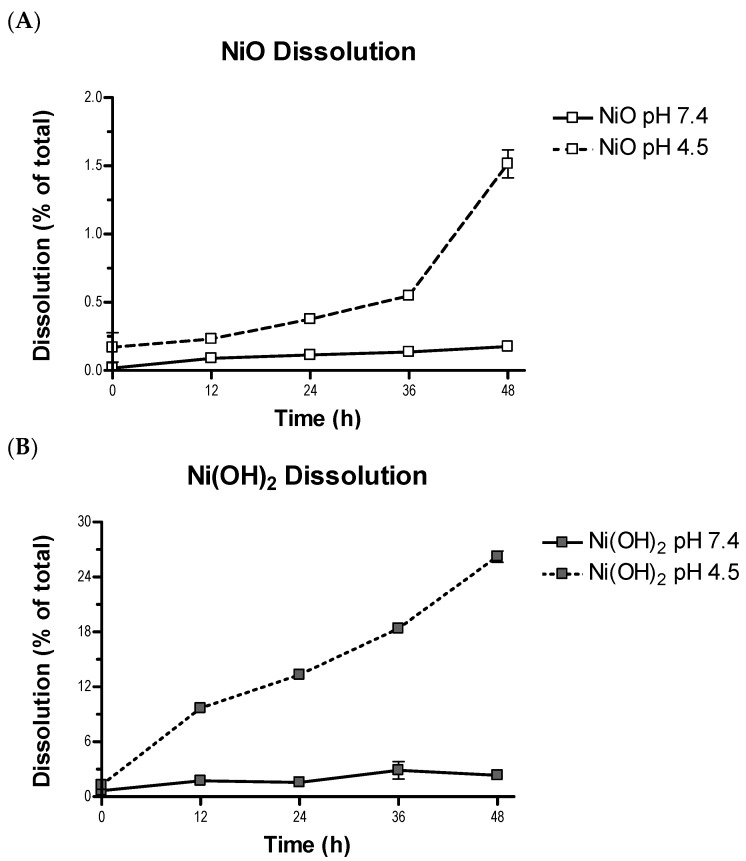
Dissolution of (**A**) NiO and (**B**) Ni(OH)_2_ at 7.4 and 4.5 pH after 12, 24, 36, and 48 h. Ni in solution was analyzed using inductively coupled plasma-optical emission spectrometry (ICP-OES) and compared to the initial mass of Ni in constant composition experiments. Values are expressed as the mean ± SD from three measurements.

The two NPs were similar in approximate physical size (APS), with a size of 15.2 ± 4.9 nm for Ni(OH)_2_ and 16.1 ± 4.8 nm for NiO. However, Ni(OH)_2_ possessed a higher specific surface area (SSA) than NiO, with Ni(OH)_2_ having an area of 103.2 m^2^/g and NiO having 73.5 m^2^/g. In our experiments, cytosolic pH conditions were considered as 7.4 pH and lysosomal conditions were considered as 4.5 pH. Ni(OH)_2_ had a larger number of relative binding sites than NiO based on X-ray photoelectron spectroscopy (XPS) analysis, with a physisorbed to chemisorbed O ratio of 1.956 at pH 7.4 and 2.018 at pH 4.5 compared to NiO with 1.245 at pH 7.4 and 0.385 at pH 4.5 (Table 2, Figure S2). Point of zero charge (PZC) analysis revealed both NiO and Ni(OH)_2_ had positive surface charge at cytosolic and lysosomal pH, with NiO at 8.7 pH and Ni(OH)_2_ at 7.9 pH, respectively (Figure 2, Table S1). Ni(OH)_2_ was more positively charged than NiO, with zeta potentials of 35.8 ± 0.7 mV and 29.1 ± 0.8 mV, respectively. Ni(OH)_2_ was also more soluble compared to NiO. The NP had a higher dissolution rate at cytosolic and lysosomal pH compared to NiO (Figure 3). The dissolution of Ni(OH)_2_ after 48 h at pH 7.4 was 2.3% and at pH 4.5 it was 26.2%. After 48 h, NiO only had a dissolution of 0.18% at pH 7.4 and 1.5% at pH 4.5. Thus, Ni(OH)_2_ had an almost 13-fold increase in dissolution at pH 7.4 compared to NiO and over a 17-fold increase at pH 4.5.

Cell culture medium was a well-buffered solution. However, to understand whether NPs can influence pH in cell culture medium, we added NPs to cell-containing medium (with supplements). The starting pH was 7.7. When incubated with cells for 24 or 48 h, with or without 100 μg/mL of NPs, the medium stayed in the range of 7.2 to 7.7 pH, which was consistent with our previous study; the slight change is negligible in consideration of cytotoxicity [20].

### 2.2. Cell Viability

**Figure 4:**
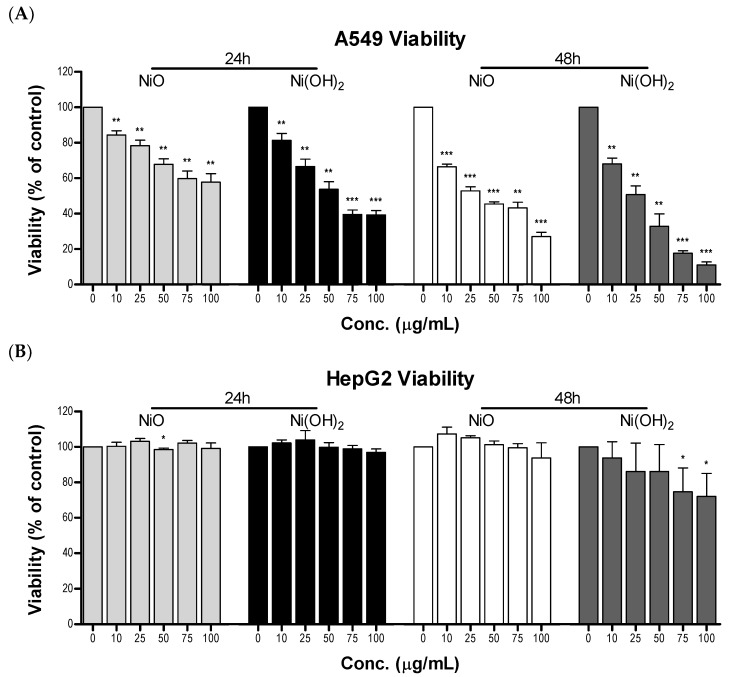
Viability of (**A**) A549 cells and (**B**) HepG2 cells upon exposure to various concentrations of NiO or Ni(OH)_2_ for 24 and 48 h. Untreated cells were normalized to 100% viable and treated cells were the percentage of viable cells compared to the control, * *p* < 0.05, ** *p* < 0.01, *** *p* < 0.001 vs. control using a one-tailed, unpaired *t*-test. Values are expressed as the mean ± SD from three independent experiments each with three trials.

A549 and HepG2 cells were treated with NiO and Ni(OH)_2_ to elucidate the cell-line specific and NP-dependent characteristics of viability. A549 cells experienced an increased loss of viability with time and concentration after exposure to both NiO and Ni(OH)_2_ (Figure 4A). Exposure for 48 h resulted in a steeper decrease in viability with increasing concentration. Each concentration tested of the NPs (0, 10, 25, 50, 75, 100 µg/mL) resulted in significantly decreased viability in A549 cells (*N* = 3, *p* < 0.05). NiO exposure of 100 μg/mL resulted in a decrease in viability of 42.2% and 73.0% after 24 and 48 h, respectively. Exposure to Ni(OH)_2_ at 100 μg/mL resulted in a 60.8% decrease in viability after 24 h and an 88.9% decrease after 48 h. The HepG2 cell line only experienced a notable decrease in viability after 48-h Ni(OH)_2_ exposure at 75 and 100 μg/mL (*N* = 3, *p* < 0.05), with a 27.9% drop in viability at 100 μg/mL (Figure 4B, Table S2). Ni(OH)_2_ resulted in a drop of 3.1% at 100 μg/mL after 24 h. NiO caused a drop of 0.8% and 6.3% after 24 and 48 h at 100 μg/mL in HepG2, respectively. A549 cells were more susceptible to the toxicity of NiO and Ni(OH)_2_ than HepG2. Ni(OH)_2_ was more toxic in both cell lines. Overall, NiO and Ni(OH)_2_ affected cell viability in a concentration-, time-, particle-, and cell line-dependent manner. Due to the significant differences in toxicity upon NiO or Ni(OH)_2_ exposure, A549 cells were subject to subsequent mechanistic studies of cytotoxicity.

### 2.3. Oxidative Stress

#### 2.3.1. Elevation of Oxidative Stress (OS)

**Figure 5:**
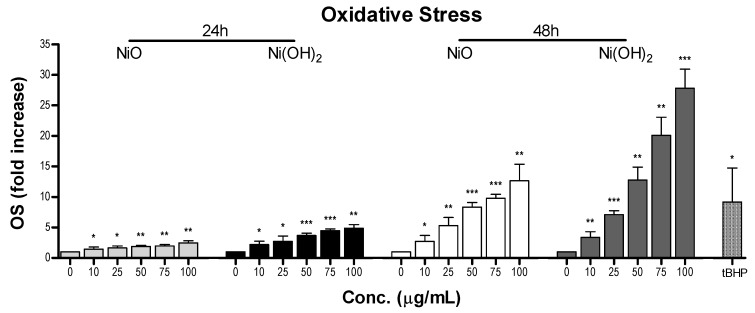
Reactive oxygen species (ROS) produced in A549 cells upon exposure to various concentrations of NiO or Ni(OH)_2_ for 24 and 48 h. Untreated cells were considered 1-fold of activity and treated cells were the relative fold increase in ROS. Tert-butyl hydroperoxide (tBHP) served as a positive control, * *p* < 0.05, ** *p* < 0.01, *** *p* < 0.001 vs. control using a one-tailed, unpaired *t*-test. Values are expressed as the mean ± SD from four independent experiments.

Oxidative stress was measured after NiO or Ni(OH)_2_ exposure for 24 and 48 h in A549 cells to determine its role in the decrease of cell viability (Figure 5). Five concentrations of each NP were tested, being 0, 10, 25, 50, 75, and 100 μg/mL. Longer exposure times resulted in a steeper increase in OS with increasing concentration of NPs. All concentrations produced OS significantly higher than the OS observed in untreated cells (*N* = 4, *p* < 0.05). A549 cells exposed to 100 μg/mL of NiO had a 2.5- and a 12.7-fold increase of OS after 24 and 48 h, respectively. Ni(OH)_2_ at 100 μg/mL caused a 4.9-fold increase in OS after 24 h and a 27.8-fold increase after 48 h. Ni(OH)_2_ induced higher levels of OS at both 24 and 48 h.

#### 2.3.2. Perturbation of Mitochondrial Membrane Potential (MMP)

**Figure 6:**
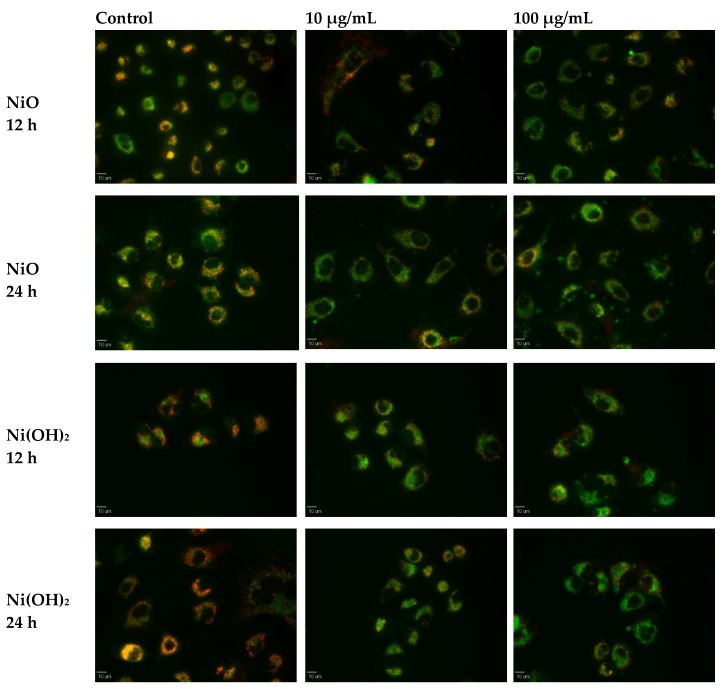
Fluorescence microscopy images of mitochondria membrane potential (MMP) after exposure to NiO or Ni(OH)_2_ for 12 or 24 h. Scale bars are 10 μm.

The dissipation of mitochondrial membrane potential was observed to determine its role in loss of viability in A549 cells upon exposure to NiO or Ni(OH)_2_ at 10 and 100 μg/mL. In the untreated control cells, an abundance of red color is indicative of healthy mitochondria (Figure 6). Cells treated with Ni(OH)_2_ or NiO experience OS and have a noticeable decrease in dysfunctional mitochondria, seen as a decrease in red color. Exposure to Ni(OH)_2_ appears to decrease the abundance of healthy mitochondria more than exposure to NiO. This is likely a result of a higher OS production upon exposure to Ni(OH)_2_, inducing a greater dissipation in MMP.

### 2.4. Apoptosis

#### 2.4.1. Elevation of Caspase-3 Enzymatic Activity

**Figure 7:**
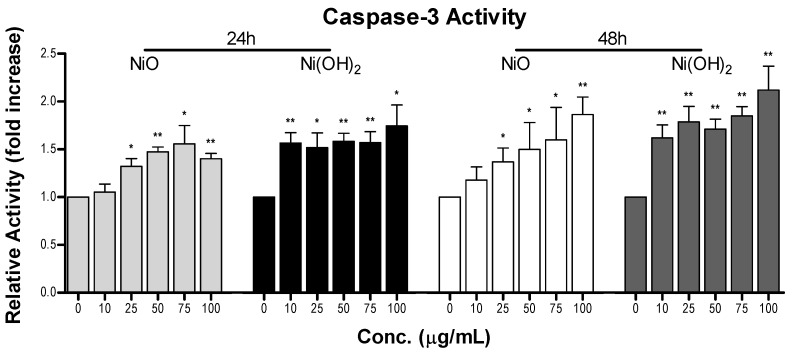
Measurement of caspase-3 activity after A549 cell exposure to various concentrations of NiO or Ni(OH)_2_ for 24 and 48 h. Untreated cells were considered 1-fold of activity and treated cells are the relative fold increase in caspase-3 activity, * *p* < 0.05, ** *p* < 0.01 vs. control using a one-tailed, unpaired *t*-test. Values are expressed as the mean ± SD from three independent experiments.

Caspase-3 enzymatic activity was measured to determine if programmed cell death was activated in A549 cells upon exposure to NiO or Ni(OH)_2_ (Figure 7). The same concentrations of NPs, 0, 10, 25, 50, 75, and 100 μg/mL, were used. Exposure to NiO significantly increased caspase-3 activity in all groups except for 10 µg/mL at 24 and 48 h (*N* = 3, *p* < 0.05). NiO exposure of 100 μg/mL caused a 1.4- and a 1.9-fold increase in caspase-3 activity at 24 and 48 h, respectively. Caspase-3 activity was significantly increased at all tested concentrations of Ni(OH)_2_ (*N* = 3, *p* < 0.05). This increase in activity was higher than NiO, with 100 μg/mL of Ni(OH)_2_ producing 1.7- and 2.2-fold increases for 24 and 48 h, respectively. Increased caspase-3 enzymatic activity for Ni(OH)_2_ compared to NiO is consistent with the increased loss of viability.

#### 2.4.2. Induction of Apoptosis

**Figure 8:**
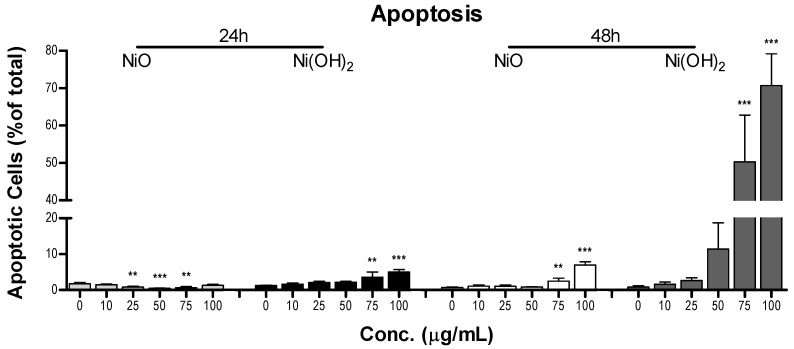
Flow cytometer analysis of total apoptosis in A549 cells after exposure to various concentrations of NiO or Ni(OH)_2_ for 24 and 48 h, ** *p* < 0.01, *** *p* < 0.001 compared to each respective control using a one-way ANOVA with a Dunnett comparison. Values are expressed as the mean ± SD from three independent experiments.

The levels of induced apoptosis were quantified using flow cytometry to elucidate programmed cell death in A549 cells when exposed to the two NPs (Figure 8 and Figure S3A,B). For our purpose, the total apoptotic percentage of each population was the summation of the subpopulations of cells undergoing early apoptosis and late apoptosis. Measurement of apoptosis in cells using a flow cytometer reflects the number of cells currently undergoing apoptosis and not total viability. NiO exposure for 24 h resulted in a statistically significant decrease in apoptosis for 25, 50, and 75 μg/mL; however, this decrease was considered negligible as the numbers were relatively low and there was no significant change in apoptosis at 100 μg/mL compared to the control. Apoptosis was increased significantly at 75 and 100 μg/mL for 48 h NiO, 24 h Ni(OH)_2_, and 48 h Ni(OH)_2_ exposure (*N* = 3, *p* < 0.01). Exposure to 100 μg/mL of NiO resulted in a 6.3% increase in apoptosis after 48 h. There was a 3.8% and a 69.9% increase in apoptosis after exposure to 100 μg/mL of Ni(OH)_2_ at 24 and 48 h, respectively.

### 2.5. Cell Cycle and Proliferation

#### 2.5.1. Suppression of Cell Proliferation

**Figure 9:**
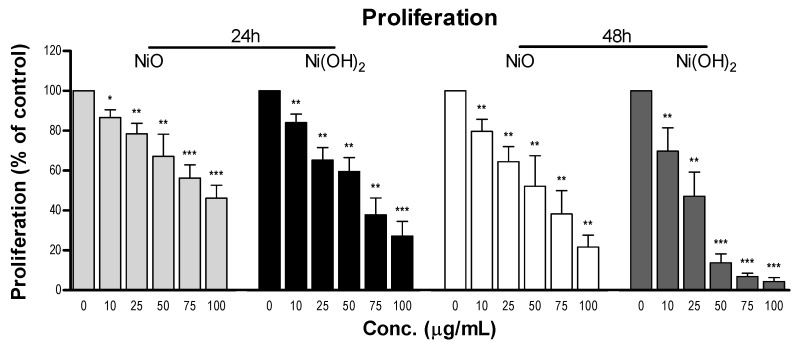
Inhibition of proliferation of A549 cells upon exposure to various concentrations of NiO or Ni(OH)_2_ for 24 and 48 h. Unexposed cells were normalized to 100% proliferative and exposed cells were the percentage of proliferating cells compared to the control, * *p* < 0.05, ** *p* < 0.01, *** *p* < 0.0001 vs. control using a one-tailed, unpaired *t*-test. Values are expressed as the mean ± SD from four independent experiments.

Proliferation was analyzed to determine proliferation’s role in A549 cell viability (Figure 9). Exposure to NiO or Ni(OH)_2_ significantly reduced the rate of proliferation at all tested concentrations at both time points (*N* = 4, *p* < 0.05). A steady decrease in proliferation was seen as concentration increased and the decrease was steeper for longer exposure. Consistent with other results, Ni(OH)_2_ produced stronger suppression of proliferation. NiO exposure of 100 μg/mL caused a decrease in cell proliferation of 53.9% and 78.4% after 24 and 48 h, respectively. Exposure to 100 μg/mL of Ni(OH)_2_ resulted in a decrease of 72.9% and 95.7% for 24- and 48-h cell proliferation, respectively.

#### 2.5.2. Alteration of Cell Cycle

**Figure 10:**
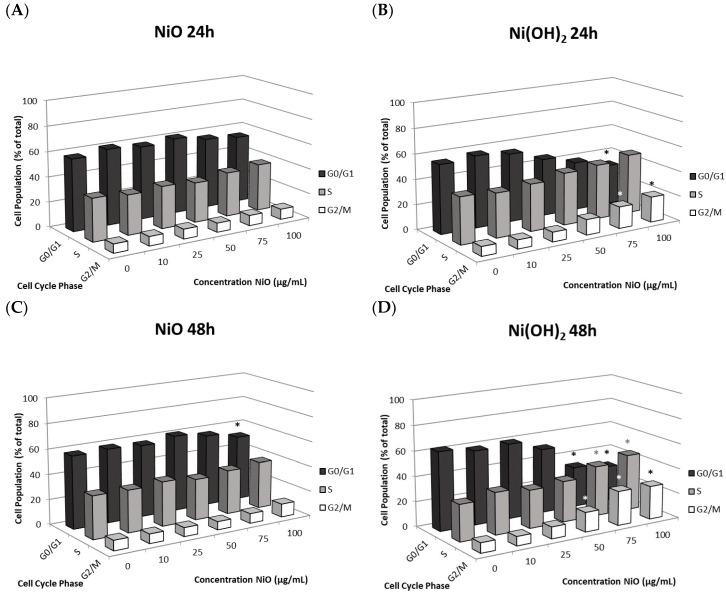
Flow cytometer analysis of cell cycle phase distribution of A549 cells. Analysis was measured after exposure to various concentrations of nanoparticle at (**A**) 24 h NiO, (**B**) 24 h Ni(OH)_2_, (**C**) 48h NiO, and (**D**) 48h Ni(OH)_2_, * *p* < 0.05 compared to each respective control using a one-way ANOVA with a Dunnett comparison. Values are expressed as the mean ± SD from three independent experiments.

The alteration of cell cycle was measured in A549 cells to determine whether cells became arrested in various phases of the cell cycle upon exposure to NiO or Ni(OH)_2_ (Figure 10). The changes induced by NiO were not significant at any concentration or at either time point, except for the decrease in G_0_/G_1_ observed at 48-h 100 μg/mL exposure (Figure 10A,B). NiO exposure of 100 μg/mL after 24 h resulted in a 3.4% decrease in G_0_/G_1_, a 3.5% increase in S, and a 1.2% increase in G_2_/M. Exposure to 100 μg/mL of NiO for 48 h resulted in a decrease of 5.8% in G_0_/G_1_, an increase of 3.4% in S, and an increase of 2.4% in G_2_/M (*N* = 3, *p* < 0.05 for G_2_/M). Exposure to Ni(OH)_2_ led to cell cycle dysregulation, with decreasing cells in G_0_/G_1_, increasing cells in S, and increasing cells in G_2_/M with increasing Ni(OH)_2_ concentration (Figure 10C,D and Figure S3C,D). Ni(OH)_2_ produced a more dramatic shift in cell cycle, with significant changes in G_0_/G_1_ at 100 μg/mL and in G_2_/M at 75 and 100 μg/mL after 24 h (*N* = 3, *p* < 0.05). G_0_/G_1_ was decreased by 23.1%, S was increased by 10.2%, and G_2_/M was increased by 12.9% for 24-h Ni(OH)_2_ 100 μg/mL exposure. After 48 h, 75 and 100 μg/mL of Ni(OH)_2_ exposure induced significant changes in G_0_/G_1_, S, and G_2_/M, with G_2_/M also experiencing a significant change at 50 μg/mL (*N* = 3, *p* < 0.05). Exposure of 100 μg/mL of Ni(OH)_2_ after 48 h produced a G_0_/G_1_ decrease of 34.1%, an S increase of 15.5%, and a G_2_/M increase of 18.6%. The increase in the proportion of cells in S and G_2_/M indicates cells are arresting in these phases. Collectively, arrest is much more prevalent and prominent when cells are treated with Ni(OH)_2_.

## 3. Discussion

In this study, we investigated and compared the cytotoxicity of two nickel NPs. Several cellular responses were explored as components of this cytotoxicity. We hypothesized that (1) the differential cytotoxicity of NiO and Ni(OH)_2_ NPs is cell line-, particle-, time-, and dose-dependent, (2) cytotoxicity is mediated by oxidative stress and subsequent cellular events including modulation of mitochondrial membrane potential and caspase-3 enzyme activity, and (3) exposure to NiO and Ni(OH)_2_ NPs alters cell cycle and suppresses cell proliferation.

Cell viability is cell line-dependent. A549 cells (a lung cell line) are much more sensitive to NPs than HepG2 cells (a liver cell line). As A549 cells are epithelial cells in a respiratory organ, it is reasonable that they would be more sensitive to particle exposure than hepatic cells, which are suited to interact with toxic compounds. Other studies are in agreement with this notion. For instance, A549 cells experienced greater induction of OS, lactate dehydrogenase leakage, reduction in glutathione levels, dissipation of MMP, elevation of apoptotic gene expression, and decline in cellular viability compared to HepG2 cells upon exposure to CuFe_2_O_4_ and ZnFe_2_O_4_ NPs [30,31]. Upon exposure to a variety of sizes and concentrations of silica NPs, HepG2 cells were less susceptible than A549 cells and exhibited a lower degree of toxic responses, including decreased ROS induction, lower decline in glutathione (GSH), and less reduction of cell viability [32]. A549 cells also experienced a greater reduction in MMP and reduction of viability than HepG2 cells upon treatment with silver NPs [33]. One possible explanation regarding the discrepancy between the in vitro toxic responses of the two cell types may be due to the fact that the liver is primarily responsible for removing toxic compounds from the body and has a higher capacity for detoxification (i.e., phase I & II enzymes) than the lung. On a different note, previously we conducted a study on comparative cytotoxicity between two lung cell lines using seven transition metal oxide nanoparticles [20]. BEAS-2B is an immortalized, but not cancerous, human bronchial epithelial cell line, whereas A549 is a human bronchoalveolar carcinoma-derived cell line. Both cell types showed similar trends of toxicity. The issue of organ-specific and cell type-specific cytotoxicity is still unsettled and deserves further attention.

We found that NiO- and Ni(OH)_2_-induced cytotoxicity is concentration-, time-, and particle-specific in A549 cells. A549 cells experienced concentration-dependent viability at all tested dosages. Ni(OH)_2_ consistently produced more severe outcomes compared to NiO and increased treatment time led to increased cytotoxic effects for both particles. Previously, the toxicity of Ni(OH)_2_ had not been examined in human cells. However, our viability results have a similar trend to a study conducted in AS52 cells, where viability was found to be concentration-dependent and the median lethal concentration (LC_50_) of Ni(OH)_2_ was found to be six times greater than that of NiO [29].

**Figure 11:**
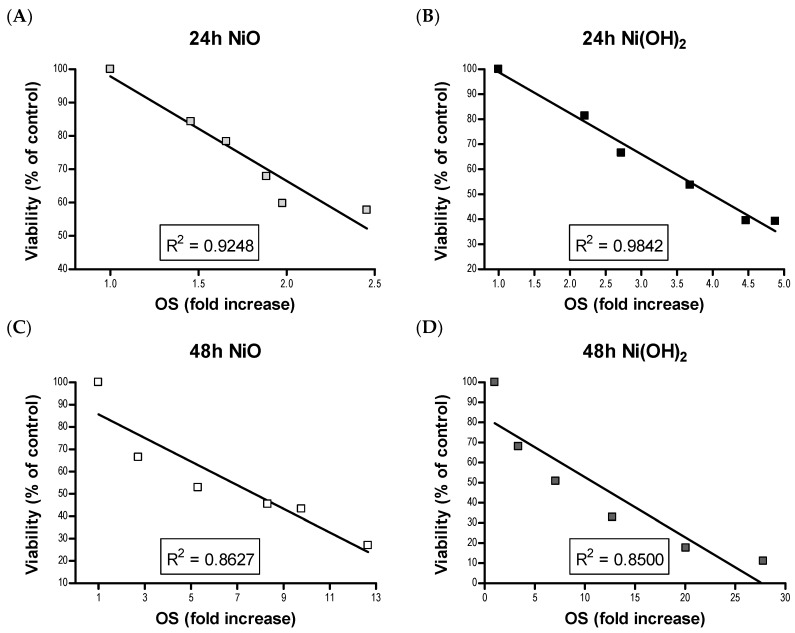
Linear correlation between viability and oxidative stress (OS) rankings for (**A**) 24 h NiO, (**B**) 24 h Ni(OH)_2_, (**C**) 48 h NiO, and (**D**) 48 h Ni(OH)_2_.

OS was elevated upon exposure to NiO and Ni(OH)_2_ and had a strong correlation with cell viability at both 24 and 48 h (Figure 11). This indicates that the generation of free radicals and oxidants is a hallmark of NP toxicity and stress from these oxidative species triggers consequential molecular events leading to cell death. OS-mediated dissipation of MMP due to the presence of both NPs was supported by the apparent reduction in influx of cationic JC-1 into mitochondria. Reduction in the number of healthy mitochondria, or their general functionality, in a cell plays a substantial role in perturbing the homeostasis of bioenergetics and multiple signaling pathways pertaining to cell survival. One such signaling alteration is the increase of caspase-3 enzymatic activity and subsequent apoptosis. Our data demonstrate that both NiO and Ni(OH)_2_ elevate caspase-3 enzymatic activity and apoptosis in a time- and concentration-dependent manner. NP-induced cell death is complex. In the present study, Ni(OH)_2_ NPs imposed a much more significant elevation of apoptosis than NiO NPs, particularly towards 48-h exposure. Besides apoptosis, necrosis is also involved in NP-induced cell death. Capasso et al. found a concentration-dependent increase of necrosis mediated by NiO NPs [21]. Another study found that CuO, ZnO, and Mn_2_O_3_ induced apoptosis in A549 cells but apoptotic cell populations increased in various increments, with the apoptotic rate staying relatively the same between two concentrations before drastically increasing in subsequent concentrations [20]. Additionally, poly vinyl pyrrolidone-coated Ag and Ag^+^ NPs induced both apoptosis and necrosis in time- and particle-dependent manners in THP-1 monocyte cells [34]. Collectively, the roles of apoptosis and necrosis seem to be dynamic in the context of acute response and prolonged exposure.

**Figure 12:**
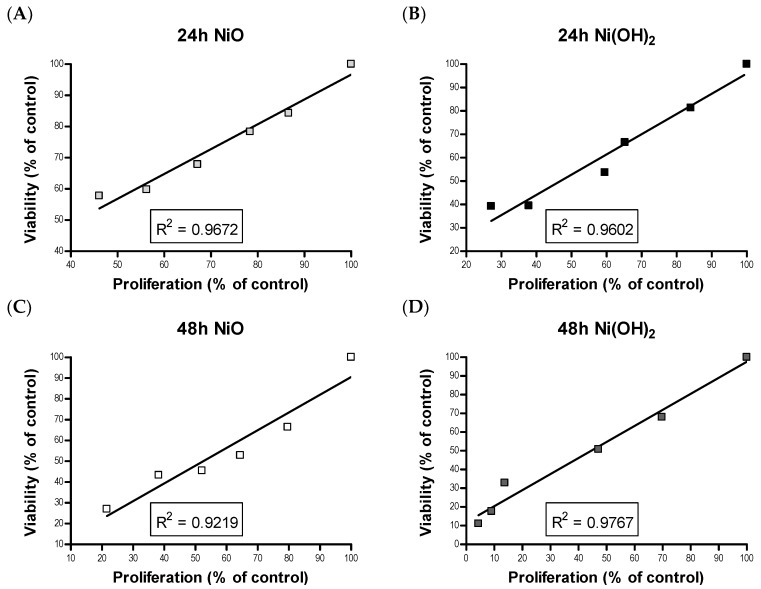
Linear correlation between viability and proliferation rankings for (**A**) 24 h NiO, (**B**) 24 h Ni(OH)_2_, (**C**) 48 h NiO, and (**D**) 48 h Ni(OH)_2_.

Although cell death induced by NPs has been demonstrated by a wealth of literature, suppression of cell proliferation is relatively under-studied. We hypothesized that the degree of cell viability imposed by exposure to NPs would be a function of cell death and cell proliferation. Our tritiated thymidine incorporation assays provided proliferation data which possessed a very strong linear correlation with cell viability for NiO and Ni(OH)_2_ over a period of 24 and 48 h (Figure 12). These correlations indicate that suppression of proliferation is a key factor in determining the reduction of cell viability. Modulation of cell proliferation has multiple causations, alteration of cell cycle being one. Our results showed that Ni(OH)_2_ arrests cells in the S and G_2_/M phases while NiO did not influence the cycle significantly. This also indicated that more factors play into proliferation rate. Although studies have found NP-mediated, phase-specific alterations of cell cycle, the effects are not all the same. For instance, exposure to TiO_2_ caused HaCat cells to arrest in the S phase while ZnO and CuO exposure caused the same cell type to arrest in G_2_/M [35,36,37]. NPs may be composed of the same elements but have different properties based on their structure, which can also influence phase-specific arrest. Spherical TiO_2_ NPs arrested A549 cells in the G_0_/G_1_ phase while needle-like TiO_2_ NPs arrested A549 cells in G_2_/M [38,39]. The mechanism of how cell cycle deregulation eventually influences cell proliferation remains to be elucidated.

Among the measured physical and chemical properties, specific surface area, metal dissolution, and surface charge are different between NiO and Ni(OH)_2_ NPs. Ni(OH)_2_ NPs have a higher specific surface area, indicative of more available binding sites (Table 2) to interact with biomolecules such as protein, lipids, and nucleic acids. Consequentially, more interactions can lead to a higher degree of observed cellular injuries. One remaining issue is identification of chemical mechanism(s) (e.g., oxidation-reduction potential between NPs and biomolecules) that may damage biomolecules. In our previous study [20], ions dissolved from transition metal oxides correlated with the differential toxicity of seven NPs. Therefore, we suspect that dissolution and the effects of ions may play a role in the observed differential toxicity between NiO and Ni(OH)_2_. Compared with NiO, a higher degree of metal dissolution from Ni(OH)_2_ might lead to Ni^2+^-mediated toxicity. It is likely that once these particles are internalized and reside in the acidic lysosomal environment, the generation and intracellular action of Ni^2+^ would be more exacerbated for Ni(OH)_2_ than NiO as Ni(OH)_2_ has been shown to have a much higher dissolution at lysosomal pH as compared to NiO (Figure 3). Both NPs possess positive surface charge, which allows for electrostatic interactions with negative molecules, such as glycosaminoglycans, leading to endocytosis [40]. The slight surface charge difference between these two NPs is not likely a key factor of cellular availability.

## 4. Materials and Methods

### 4.1. Material Sources

NiO NPs were purchased from Nanostructured and Amorphous Materials (Los Alamos, Houston, TX, USA) and Ni(OH)_2_ NPs were purchased from US Research Nanomaterials (Houston, TX, USA). A549 cells and HepG2 cells were acquired from the American Tissue Culture Collection (Manassas, VA, USA). 2′,7′-dichlorodihydrofluorescein diacetate (H_2_DCFDA) and propidium iodide (PI) were obtained from Fisher Scientific (St. Peters, MO, USA). The JC-1 Mitochondrial Membrane Potential Detection Kit and sulforhodamine B were purchased from Biotium (Freemont, CA, USA). Ac-DEVD-pNA was obtained from Anaspec (Fremont, CA, USA). Annexin V-FITC and 7-aminoactinomycin D (7-AAD) were acquired from BD Biosciences (Franklin Lakes, NJ, USA). Tritiated thymidine was purchased from Perkin-Elmer (Waltham, MA, USA). Other chemicals used for experiments were of the highest purity that could be obtained.

### 4.2. Storage and Characterization of Nanoparticles

NPs were stored in an amber desiccator under a pure nitrogen atmosphere to protect them from moisture, oxidation, and UV damage. The instrumentation and protocols used to characterize NPs followed our previous publication [20]. SSA and APS of NPs in non-aqueous conditions were measured by Brunauer–Emmett–Teller (BET) and TEM, respectively. Morphology and crystal structure, surface charge, metal dissolution, and relative available surface binding sites of NPs in aqueous conditions were measured by high resolution transition electron microscopy (HRTEM), PZC, inductively coupled plasma-optical emission spectrometry (ICP-OES), and XPS.

### 4.3. TEM and HRTEM

NiO and Ni(OH)_2_ NPs were suspended in ethanol and dropped onto copper grids with amorphous carbon film. Grids were allowed to dry overnight before insertion into a Tecnai F20 TEM (Thermo Fisher Scientific, Hillsboro, OR, USA) equipped with an energy-dispersive detector (EDS) detector. TEM and HRTEM images were captured, as well as the SAED patterns for both samples.

### 4.4. Quantification of Available Binding Sites

XPS integrated peak areas of the O 1s binding energy core level were used to quantify the relative number of available binding sites for material in the extracellular matrix to interact with on the NiO and Ni(OH)_2_ nanoparticle surfaces. Quantification was achieved by noting the physisorbed versus chemisorbed oxygen on the NP surfaces as described in our previous study [20]. In the case of NiO NPs, the deconvoluted O 1s peak denoting the metal oxide represents the underlying substrate while those peak areas of those oxidation states not from the metal oxide denote physisorbed O. The number of available binding sites were quantified by dividing the XPS peak area of the physisorbed O by that of chemisorbed O.

The O 1s core levels of the NiO NPs were observed at 529.0 eV and 531.0 eV, denoting the metal oxide [41,42,43] and physisorbed O on the NP surfaces, respectively. For the Ni(OH)_2_ NP surfaces, the binding energy centers of the O 1s orbitals appear at 531.2 and 532.9 eV, denoting chemisorbed OH groups and physisorbed OH/H_2_O [41,44] on the NP surfaces, respectively. While binding energies for adsorbed OH/H_2_O are well established, they are not for chemisorbed OH on Ni(OH)_2_; this information was obtained via XPS scans on the dried Ni(OH)_2_ NPs performed in our laboratory as a reference standard. The binding energy peak center for this oxidation state was found to be 531.2 eV, having a full width at half maximum (FWHM) of 3.4 km/sec, the parameters of which were used in peak area deconvolution of the O 1s envelopes.

### 4.5. Cell Culture and Nanoparticle Treatment

A549 cells were maintained in Ham’s F-12 modified medium supplemented with 10% HyClone FetalClone serum (GE Healthcare Life Sciences, Marlborough, MA, USA) and 1% penicillin/streptomycin. HepG2 cells were maintained in Eagle’s minimum essential medium supplemented with 10% FetalClone serum and 1% penicillin/streptomycin. Both cell lines were grown in 10-cm tissue culture dishes at 37 °C in a 5% CO_2_ humidified incubator. Upon reaching a confluence of ca. 70–80%, cells were trypsinized, and appropriate numbers of cells were seeded into tissue culture dishes or plates for various experiments. NPs were suspended in cell culture media to create a working concentration of 1 mg NP per 1 mL media. The working suspension of NPs was sealed with parafilm and sonicated for 3 min to break up aggregates. The suspension was vortexed to achieve a homogenous mixture before being added to cells and was diluted in cellular media to achieve a series of desired concentrations. Each treatment group was completed in at least triplicate and appropriate controls were included, with the most common controls being untreated cells.

### 4.6. Cell Viability

Cell viability was measured using the sulforhodamine B (SRB) assay. A549 cells and HepG2 cells were seeded into 24-well tissue culture plates and allowed to grow for 24 h before compound exposure. For A549 cells, 45,000 and 22,000 cells were seeded per well for 24- and 48-h exposure, respectively. For HepG2 cells, 120,000 cells were seeded per well for both 24 and 48 h. Cells were treated with nickel NPs (0, 10, 25, 50, 75, or 100 µg/mL) for 24 or 48 h. Upon termination of experiments, cell medium was discarded from the cells. The cells were fixed with cold 10% trichloroacetic acid (TCA) for 1 h at 4 °C. The fixed cells were then washed three times with distilled water and then allowed to dry completely. Cells were incubated with 0.5 mL of SRB staining solution (0.2% SRB in 1% acetic acid) for 30 min at room temperature. The cells were then washed three times with 1 mL of 1% acetic acid for 20 min on a rocker to eliminate excess dye. A Q-tip was used to remove excess solution stuck to the sides of the wells. Acetic acid removal was followed by addition of 400 µL of cold 10 mM Tris-HCl solution to each well for 20 min. Aliquots of 250 μL each were transferred onto a 96-well plate and absorbance was measured at 510 nm using a microplate reader (FLUOstar Omega, BMG Labtechnologies, Cary, NC, USA). Cell viability of treatment groups was calculated based on the percent absorbance relative to the control group with appropriate blanks subtracted.

### 4.7. Oxidative Stress (OS)

Reactive oxidative species were measured with H_2_DCFDA. Upon entry of cells, H_2_DCFDA was deacetylated by esterases to a non-fluorescent compound. When H_2_DCFDA is oxidized by reactive oxidative species, it is converted to the highly fluorescent compound 2′,7′-dichlorofluorescein (DCF) and can be detected by fluorescence spectroscopy. A549 cells were exposed to a series of concentrations of NPs (0, 10, 25, 50, 75, or 100 µg/mL) for 24 or 48 h. Cells were seeded into 96-well plates at 1500 or 750 cells per well for 24- and 48-h treatments, respectively, and grown for 24 h before exposure. As a positive control, cells were incubated with 400 µM tert-butyl hydroperoxide (tBHP) at 37 °C for 1 h before termination of the experiment. Upon termination, the media was removed from the cells followed by a wash with phosphate-buffered saline (PBS). Eighty µL of 0.87 mM H_2_DCFDA in ethanol was added to each well and the plate was incubated for 1 h. Cells were then washed with PBS three times followed by addition of 100 µL of PBS. Fluorescence was measured at 510 nm using a microplate reader. The florescence intensity of cells in experimental plates was divided by the fluorescence intensity of the control group to determine the percent increase in ROS with appropriate blank intensities considered.

### 4.8. Mitochondrial Membrane Potential

Mitochondrial membrane potential was determined with fluorescence microscopy using the JC-1 MMP Detection Kit (Cayman Chemical Company, Ann Arbor, MI, USA). JC-1 (5,5′,6,6′-Tetrachloro-1,1′,3,3′-tetraethylbenzimidazolylcarbocyanine iodide, CAS#: 3520-43-2) monomers fluoresce green when in the cytosol of cells. Accumulation of JC-1 in mitochondria allows the compound to form aggregates that exhibit red fluorescence in a concentration-dependent manner. Normal mitochondrial membrane potential, in healthy cells, allows JC-1 to influx and form aggregates in the mitochondria. In unhealthy cells, mitochondrial membrane potential is decreased, JC-1 concentration cannot increase high enough to form aggregates, and thus the compound remains green [45]. The comparison of red and lack of red fluorescence allows the loss of mitochondrial membrane potential to be observed.

A549 cells were grown for 24 h in 35-mm glass-bottom culture dishes at 15,000 cells per dish. Cells were exposed to several concentrations of nickel NPs (0, 10, or 100 µg/mL) for 12 or 24 h. Following NP treatment, the plates were incubated with 100 µL of JC-1 staining solution per mL of medium at 37 °C for 15 min. Each plate was then washed with 1 mL of PBS followed by addition of 1 mL of PBS before fluorescence detection under an epifluorescence microscope (Olympus Corporation, Tokyo, Japan). Red fluorescence was observed with a Texas Red filter (excitation/emission: 590/610 nm) while green fluorescence was with a FITC filter (excitation/emission: 490/520 nm).

### 4.9. Caspase-3 Activity

Caspase-3 enzymatic activity was measured using Ac-DEVD-pNA as a substrate. A549 cells were grown for 24 h in 24-well plates at seeding densities of 45,000 or 22,000 cells per well for 24- or 48-h exposure, respectively. Cells were treated with a series of concentrations of NPs (0, 10, 25, 50, 75, or 100 µg/mL) for 24 or 48 h. After incubation with NPs, cells were washed once with 0.5 mL of PBS. Two hundred µL of cold lysis buffer (50 mM Tris-HCl, 1.5 mL of 5 M NaCl, 0.25 g of sodium deoxycholate, 1 mM ethylenediaminetetraacetic acid (EDTA), 0.5 mL of Triton-100, and 50 mL of distilled water) was added to each well, cells were scrapped off the well bottoms, resuspended in the lysis buffer, and incubated at 4 °C for 10 min. Samples were centrifuged at 15,000× *g* for 20 min at 4 °C. Total protein in each sample was measured using a Pierce BCA protein assay (Thermo Scientific, Rockford, IL, USA). Cell lysate and reaction buffer (20% glycerol, 0.5 mM EDTA, 5 mM dithiothreitol, and 100 mM 4-(2-hydroxyethyl)-1-piperazineethanesulfonic acid (HEPES), pH 7.5) were combined in a 96-well plate to have 20 µg of cellular protein and a total volume of 198 µL in each well. Following, 2 µL of 0.5 mg/mL Ac-DEVD-pNA substrate was added to each well. Samples were incubated at 37 °C for 6 h. Absorbance of enzyme-catalyzed release of p-nitroanilide was measured at 405 nm with a microplate reader.

### 4.10. Apoptosis

Apoptosis was measured with flow cytometry using annexin V-FITC and 7-AAD. A549 cells were seeded into 6-cm tissue culture dishes at densities of 250,000 and 120,000 for 24- and 48-h exposure, respectively, and allowed to grow for 24 h before treatment. Cells were treated with a series of NP concentrations (0, 10, 25, 50, 75, or 100 µg/mL) for 24 or 48 h. Upon termination of the NP exposure period, cells were washed with PBS and harvested with trypsinization. NP treatment medium, PBS washes, and trypsinized cells were all collected in the same centrifuge tube for each concentration of NP in order to avoid loss of floating cells undergoing apoptosis. The samples were centrifuged and the supernatant was discarded. The pellet was washed with 1 mL of ice-cold PBS, centrifuged again, and the supernatant was removed. The cells were resuspended in 100 µL of 1x annexin V binding buffer, 2 µL of annexin V-FITC, and 2 µL of 7-AAD. The cells were then incubated for 20 min in the dark. The stained cell solutions were transferred to the wells of a 96-well plate for flow cytometry analysis on a CytoFLEX flow cytometer (Beckman-Coulter, Brea, CA, USA). Cells in different stages of apoptosis were quantified using FCS Express 6 (DeNovo software, Pasadena, CA, USA). Early and late apoptotic cells were added to represent the total percentage of apoptotic cells.

### 4.11. Cell Proliferation

Proliferation was determined with a tritiated thymidine ([5′-^3^H]-thymidine) incorporation assay. A549 cells were seeded in 24-well plates with 45,000 and 22,000 cells per well for 24- and 48-h exposure, respectively. Cells were grown for 24 h before being dosed with nickel compounds. Cells were exposed to a series of concentrations of NPs (0, 10, 25, 50, 75, or 100 µg/mL) and treated with [5′-^3^H]-thymidine (Perkin-Elmer) simultaneously for 24 or 48 h. A working solution of [5′-^3^H]-thymidine was prepared with 20 µL of [5′-^3^H]-thymidine (1 µCi/µL) in 500 µL of PBS. Each well of the 24-well plate was treated with 20 µL of the [5′-^3^H]-thymidine working solution. Upon termination of each experiment, cells were washed twice with ice-cold PBS. The cells were then fixed in 0.5 mL of ice-cold 10% TCA for 5 min on ice. TCA fixation was repeated once. Cells were brought to room temperature and lysed using 0.5 mL of room temperature 1 N NaOH for 5 min. The solution was neutralized by adding an equal amount of 1 N HCl. The lysed cell solution was thoroughly mixed by pipetting up and down and then transferred to liquid scintillation counting vials with 4 mL of Econo-Safe scintillation counting fluid (Research Products International, Mt. Prospect, IL, USA). Sample vials were then subjected to scintillation counting using a Beckman liquid scintillation counter LS6500 (Beckman-Coulter). The total count of radioactivity was divided by the radioactivity from the 0 µg/mL control cells to determine the percentage of proliferating cells compared to cells not exposed to nickel compounds. All radioactive waste was disposed of following Missouri S&T’s Department of Environmental Health and Safety procedures.

### 4.12. Cell Cycle

Alteration of cell cycle due to nickel NP exposure was measured with flow cytometry and PI staining. A549 cells were grown in 6-cm tissue culture dishes for 24 h before treatment. The seeding density of cells per dish was 250,000 cells for 24-h treatment and 120,000 cells for 48-h treatment. Cells were exposed to a series of concentrations of NPs (0, 10, 25, 50, 75, or 100 µg/mL) for 24 or 48 h. Following incubation with NP treatments, cells were washed with PBS, harvested using trypsinization, and centrifuged. The cell pellet was then resuspended in 1 mL of PBS and 3 mL of ice-cold absolute methanol was added drop-wise to the solution while vortexing. The cells were stored at 4 °C for at least 24 h to fix. After fixation, the cells were centrifuged and washed once with PBS. The cells were then suspended in PI staining solution (50 µg/mL PI, 0.1% RNase A, and 0.05% Triton X-100 in PBS) for 20 min in the dark. One mL of PBS was added to each sample before centrifuging, the supernatant was removed, and cells were resuspended in 250 µL of PBS. The stained samples were then pipetted into a 96-well plate and analyzed with a flow cytometer. FCS Express 6 was used to determine the distribution of cells in different cell cycle phases. The number of cells in each phase of the cell cycle (G_0_/G_1_, S, and G_2_/M) was totaled and the percentage in each phase was calculated.

### 4.13. Statistical Analysis

Each experiment was repeated at least three times independently with each treatment group having at least triplicate samples. Data are presented as mean ± standard deviation. Statistical analysis was performed in Minitab 19. One-tailed unpaired t-tests were used to compare experimental groups to the control group in normalized data sets, with µ > control or µ < control depending on the experimental hypothesis. Analysis of variance (ANOVA) with Dunnett comparison was used to determine significant differences against the control group. Significance was set at *p* < 0.05. *P*-values less than 0.05, 0.01, and 0.001 are noted in figure legends. Linear regression to analyze correlations between data was completed using GraphPad Prism 4. All figures were produced using GraphPad Prism 4 except for the cell cycle distribution graphs, which were produced by Microsoft Excel 2016.

## 5. Conclusions

**Figure 13:**
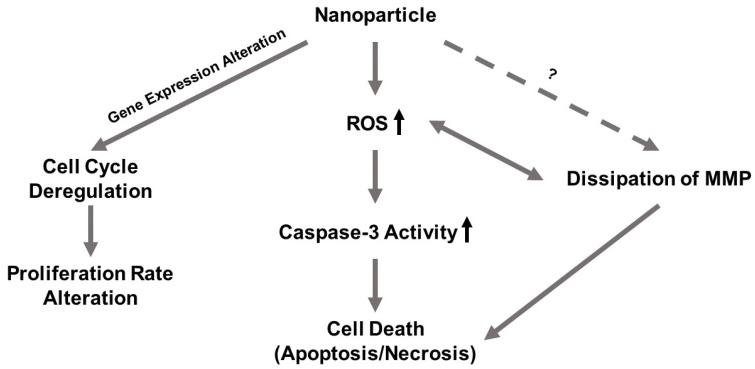
Cell viability is a function of cell death and suppression of proliferation.

Figure 13 depicts the interwoven pathways of toxic events for NPs. Toxicity exerted by NiO and Ni(OH)_2_ NPs is cell line-, concentration-, time-, and particle-dependent in the range of 10 to 100 µg/mL. Ni(OH)_2_ is more cytotoxic than NiO. NP-induced oxidative stress triggered subsequent dissipation of mitochondrial membrane potential and induction of caspase-3 enzyme activity. The subsequent apoptotic events led to the reduction of cell number. In addition to cell death, cell cycle deregulation and suppression of cell proliferation also regulated cell number. Thus, the observed cell viability is a function of cell death and suppression of proliferation. Differences in physical and chemical properties of the NPs, such as metal dissolution and total surface area, are in agreement with the observed differential toxicity of these two NPs.

## Figures and Tables

**Table 1:** Physical characteristics of NiO and Ni(OH)_2_ NPs. Data are expressed as mean ± SD.

Characteristic	NiO	Ni(OH)_2_
APS * (nm)	16.1 ± 4.8	15.2 ± 4.9
SSA ** (m^2^/g)	73.5	103.2
Apparent morphology	Aggregated nanograins	Flakey aggregate
PZC *** (pH)	8.7	7.9
Zeta potential (mV)	29.1 ± 0.8	35.8 ± 0.7

* APS, approximate physical size, ** SSA denotes specific surface area. *** PZC, point of zero charge is defined as the pH value at which the surface is electrostatically neutral.

**Table 2:** Relative number of binding sites from integrated X-ray photoelectron spectroscopy (XPS) O 1s peak areas.

NP Condition	Metal Oxide or Chemisorbed OH	Physisorbed O	Physisorbed to Chemisorbed O Ratio
NiO pH 7.4	912	1135	1.245
NiO pH 4.5	4874	1875	0.385
Ni(OH)_2_ pH 7.4	1330	2601	1.956
Ni(OH)_2_ pH 4.5	2020	4076	2.018

## Supplementary Materials

Supplementary materials can be found at https://www.mdpi.com/1422-0067/21/7/2355/s1, Figure S1: XRD spectra of NiO and Ni(OH)2. Figure S2: XPS core levels of O 1s orbitals of NiO and Ni(OH)2. Figure S3: Exemplary flow cytometer data in FCS Express 6. Table S1: Point of Zero Charge. Table S2: HepG2 Viability.

## Abbreviations

NP	Nanoparticle
APS	Approximate physical size
SSA	Specific surface area
PZC	Point of zero charge
HRTEM	High-resolution transmission electron microscopy
EDX	Energy disperse X-ray spectroscopy
XRD	X-ray diffraction
SAED	Selected area electron diffraction
XPS	X-ray photoelectron spectroscopy
BET	Brunauer–Emmett–Teller
ICP-OES	Inductively coupled plasma-optical emission spectrometry
OS	Oxidative stress
MMP	Mitochondrial membrane potential
SRB	Sulforhodamine B
7-AAD	7-aminoactinomycin D
DCF	2′,7′-dichlorofluorescein
PI	Propidium iodide

## References

[1] Nohynek G.J., Dufour E.K., Roberts M.S. (2008). Nanotechnology, cosmetics and the skin: Is there a health risk?. Skin Pharmacol. Physiol..

[2] De Jong W.H., Borm P.J. (2008). Drug delivery and nanoparticles:applications and hazards. Int. J. Nanomed..

[3] O’Neal D.P., Hirsch L.R., Halas N.J., Payne J.D., West J.L. (2004). Photo-thermal tumor ablation in mice using near infrared-absorbing nanoparticles. Cancer Lett..

[4] Kowshik M., Deshmukh N., Vogel W., Urban J., Kulkarni S.K., Paknikar K.M. (2002). Microbial synthesis of semiconductor CdS nanoparticles, their characterization, and their use in the fabrication of an ideal diode. Biotechnol. Bioeng..

[5] Weir A., Westerhoff P., Fabricius L., Hristovski K., von Goetz N. (2012). Titanium dioxide nanoparticles in food and personal care products. Environ. Sci. Technol..

[6] Li Y., Wu Y., Ong B.S. (2005). Facile synthesis of silver nanoparticles useful for fabrication of high-conductivity elements for printed electronics. J. Am. Chem. Soc..

[7] Inshakova E., Inshakov O. (2017). World market for nanomaterials: Structure and trends. MATEC Web of Conferences.

[8] Van Broekhuizen P., van Broekhuizen F., Cornelissen R., Reijnders L. (2012). Workplace exposure to nanoparticles and the application of provisional nanoreference values in times of uncertain risks. J. Nanopart Res..

[9] Lai X., Wei Y., Zhao H., Chen S., Bu X., Lu F., Qu D., Yao L., Zheng J., Zhang J. (2015). The effect of Fe2O3 and ZnO nanoparticles on cytotoxicity and glucose metabolism in lung epithelial cells. J. Appl. Toxicol..

[10] Di Bucchianico S., Gliga A.R., Akerlund E., Skoglund S., Wallinder I.O., Fadeel B., Karlsson H.L. (2018). Calcium-dependent cyto- and genotoxicity of nickel metal and nickel oxide nanoparticles in human lung cells. Part Fibre Toxicol..

[11] Vamanu C.I., Cimpan M.R., Hol P.J., Sornes S., Lie S.A., Gjerdet N.R. (2008). Induction of cell death by TiO2 nanoparticles: Studies on a human monoblastoid cell line. Toxicol. Vitro.

[12] Han J.W., Gurunathan S., Jeong J.K., Choi Y.J., Kwon D.N., Park J.K., Kim J.H. (2014). Oxidative stress mediated cytotoxicity of biologically synthesized silver nanoparticles in human lung epithelial adenocarcinoma cell line. Nanoscale Res. Lett..

[13] Limbach L.K., Li Y., Grass R.N., Brunner T.J., Hintermann M.A., Muller M., Gunther D., Stark W.J. (2005). Oxide nanoparticle uptake in human lung fibroblasts: Effects of particle size, agglomeration, and diffusion at low concentrations. Environ. Sci. Technol..

[14] Martindale J.L., Holbrook N.J. (2002). Cellular response to oxidative stress: Signaling for suicide and survival. J. Cell. Physiol..

[15] Huang Y.W., Cambre M., Lee H.J. (2017). The Toxicity of Nanoparticles Depends on Multiple Molecular and Physicochemical Mechanisms. Int. J. Mol. Sci..

[16] Jiang J., Oberdorster G., Elder A., Gelein R., Mercer P., Biswas P. (2008). Does nanoparticle activity depend upon size and crystal phase?. Nanotoxicology.

[17] Forest V., Leclerc L., Hochepied J.F., Trouve A., Sarry G., Pourchez J. (2017). Impact of cerium oxide nanoparticles shape on their In Vitro cellular toxicity. Toxicol. Vitro.

[18] Baek M., Kim M.K., Cho H.J., Lee J.A., Yu J., Chung H.E., Choi S.J. (2011). Factors influencing the cytotoxicity of zinc oxide nanoparticles: Particle size and surface charge. J. Phys. Conf. Ser.

[19] Kai W., Xiaojun X., Ximing P., Zhenqing H., Qiqing Z. (2011). Cytotoxic effects and the mechanism of three types of magnetic nanoparticles on human hepatoma BEL-7402 cells. Nanoscale Res. Lett..

[20] Chusuei C.C., Wu C.H., Mallavarapu S., Hou F.Y., Hsu C.M., Winiarz J.G., Aronstam R.S., Huang Y.W. (2013). Cytotoxicity in the age of nano: The role of fourth period transition metal oxide nanoparticle physicochemical properties. Chem. Biol. Interact..

[21] Capasso L., Camatini M., Gualtieri M. (2014). Nickel oxide nanoparticles induce inflammation and genotoxic effect in lung epithelial cells. Toxicol. Lett..

[22] AZoNano AZoNano. Nickel Oxide (NiO) Nanoparticles—Properties, Applications. https://www.azonano.com/article.aspx?ArticleID=3378.

[23] Uğurlu B. Nickel Hydroxide Nanoparticles and Usage Areas of Nickel Hydroxide Nanopowders. https://nanografi.com/blog/nickel-hydroxide-nanoparticles-and-usage-areas-of-nickel-hydroxide-nanopowders/.

[24] Cempel M., Nikel G. (2006). Nickel: A Review of its sources and environmental toxicology. Pol. J. Environ. Stud..

[25] Kadoya C., Ogami A., Morimoto Y., Myojo T., Oyabu T., Nishi K., Yamamoto M., Todoroki M., Tanaka I. (2012). Analysis of bronchoalveolar lavage fluid adhering to lung surfactant. Experiment on intratracheal instillation of nickel oxide with different diameters. Ind. Health.

[26] Kang G.S., Gillespie P.A., Chen L.C. (2011). Inhalation exposure to nickel hydroxide nanoparticles induces systemic acute phase response in mice. Toxicol. Res..

[27] Horie M., Fukui H., Nishio K., Endoh S., Kato H., Fujita K., Miyauchi A., Nakamura A., Shichiri M., Ishida N. (2011). Evaluation of acute oxidative stress induced by NiO nanoparticles In Vivo and In Vitro. J. Occup. Health.

[28] Siddiqui M.A., Ahamed M., Ahmad J., Majeed Khan M.A., Musarrat J., Al-Khedhairy A.A., Alrokayan S.A. (2012). Nickel oxide nanoparticles induce cytotoxicity, oxidative stress and apoptosis in cultured human cells that is abrogated by the dietary antioxidant curcumin. Food Chem. Toxicol..

[29] Fletcher G.G., Rossetto F.E., Turnbull J.D., Nieboer E. (1994). Toxicity, uptake, and mutagenicity of particulate and soluble nickel compounds. Environ. Health Perspect..

[30] Alhadlaq H.A., Akhtar M.J., Ahamed M. (2015). Zinc ferrite nanoparticle-induced cytotoxicity and oxidative stress in different human cells. Cell Biosci..

[31] Ahmad J., Alhadlaq H.A., Alshamsan A., Siddiqui M.A., Saquib Q., Khan S.T., Wahab R., Al-Khedhairy A.A., Musarrat J., Akhtar M.J. (2016). Differential cytotoxicity of copper ferrite nanoparticles in different human cells. J. Appl. Toxicol..

[32] Kim I.Y., Joachim E., Choi H., Kim K. (2015). Toxicity of silica nanoparticles depends on size, dose, and cell type. Nanomedicine.

[33] Xin L., Wang J., Fan G., Che B., Wu Y., Guo S., Tong J. (2016). Oxidative stress and mitochondrial injury-mediated cytotoxicity induced by silver nanoparticles in human A549 and HepG2 cells. Environ. Toxicol..

[34] Foldbjerg R., Olesen P., Hougaard M., Dang D.A., Hoffmann H.J., Autrup H. (2009). PVP-coated silver nanoparticles and silver ions induce reactive oxygen species, apoptosis and necrosis in THP-1 monocytes. Toxicol. Lett..

[35] Gao X., Wang Y., Peng S., Yue B., Fan C., Chen W., Li X. (2015). Comparative toxicities of bismuth oxybromide and titanium dioxide exposure on human skin keratinocyte cells. Chemosphere.

[36] Gao F., Ma N., Zhou H., Wang Q., Zhang H., Wang P., Hou H., Wen H., Li L. (2016). Zinc oxide nanoparticles-induced epigenetic change and G2/M arrest are associated with apoptosis in human epidermal keratinocytes. Int. J. Nanomed..

[37] Luo C., Li Y., Yang L., Zheng Y., Long J., Jia J., Xiao S., Liu J. (2014). Activation of Erk and p53 regulates copper oxide nanoparticle-induced cytotoxicity in keratinocytes and fibroblasts. Int. J. Nanomed..

[38] Moschini E., Gualtieri M., Gallinotti D., Pezzolato E., Fascio U., Camatini M., Mantecca P. (2010). Metal oxide nanoparticles induce cytotoxic effects on human lung epithelial cells A549. Chem. Eng. Trans..

[39] Wang Y., Cui H., Zhou J., Li F., Wang J., Chen M., Liu Q. (2015). Cytotoxicity, DNA damage, and apoptosis induced by titanium dioxide nanoparticles in human non-small cell lung cancer A549 cells. Environ Sci Pollut Res Int.

[40] Gump J.M., June R.K., Dowdy S.F. (2010). Revised role of glycosaminoglycans in TAT protein transduction domain-mediated cellular transduction. J. Biol. Chem..

[41] Khawaja E.E., Salim M.A., Khan M.A., Al-del F.F., Khattak G.D., Hussain Z. (1989). XPS, auger, electrical and optical studies of vanadium phosphate glasses doped with nickel oxide. J. Non Cryst. Solids.

[42] Venezia A.M., Bertoncello R., Deganello G. (1995). X-ray photoelectron investigation of pumice-supported nickel catalysts. Surf. Interface Anal..

[43] Lian K., Thorpe S.J., Kirk D.W. (1992). Electrochemical and surface characterization of electrocatalytically active amorphous Ni CO alloys. Electrochim. Acta.

[44] Wagner C.D., Zatka D.A., Raymond R.H. (1980). Use of the oxygen KLL Auger lines in identification of surface chemical states by electron spectroscopy for chemical analysis. Anal. Chem..

[45] Sivandzade F., Bhalerao A., Cucullo L. (2019). Analysis of the mitochondrial membrane potential using the cationic JC-1 Dye as a sensitive fluorescent probe. Bio. Protoc..

