# Supplementary material for: Cytotoxicity of NiO and Ni(OH)2 Nanoparticles Is Mediated by Oxidative Stress-Induced Cell Death and Suppression of Cell Proliferation
Source: Int J Mol Sci. 2020 Mar 28;21(7):2355. doi: 10.3390/ijms21072355 (PMC7178005; doi:10.3390/ijms21072355)
Supplement: Supplementary file 1 [file ijms-21-02355-s001.pdf]

International Journal of
Molecular Sciences

MDPI

Supplementary

Cytotoxicity of NiO and Ni(OH)2 Nanoparticles is Mediated by Oxidative Stress-induced Cell Death and Suppression of Cell Proliferation

Melissa H. Cambre [1,†], Natalie J. Holl [1,†], Bolin Wang [1], Lucas Harper [1], Han-Jung Lee [2], Charles C. Chusuei [3], Fang Yao Stephen Hou [4], Ethan T. Williams [3], Jerry D. Argo [3], Raja Ram Pandey [3] and Yue-Wern Huang [1,*]

(A)

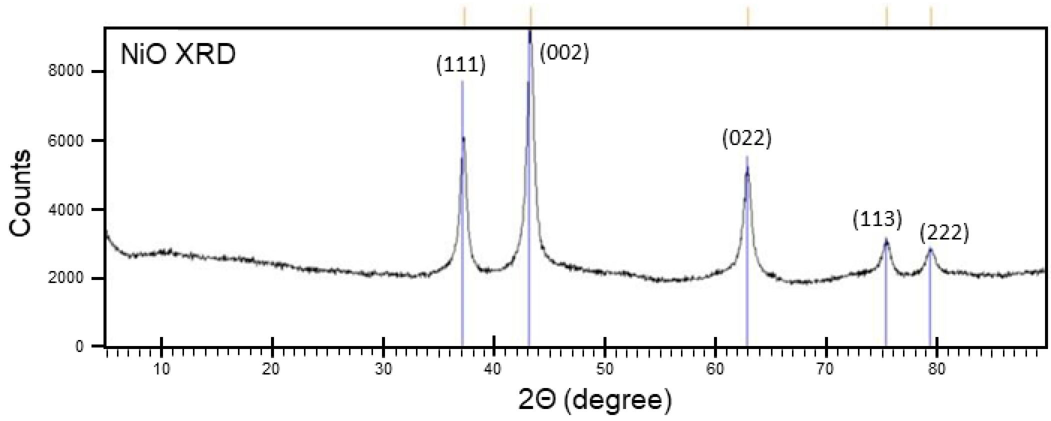

(B)

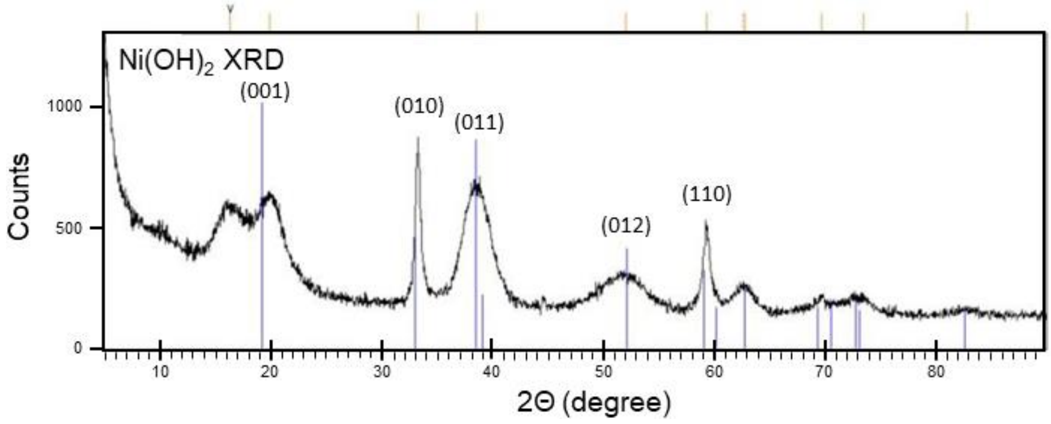

Figure S1. XRD spectra of (**A**) NiO and (**B**) Ni(OH)$_2$, with the (hkl) indicated above each peak.

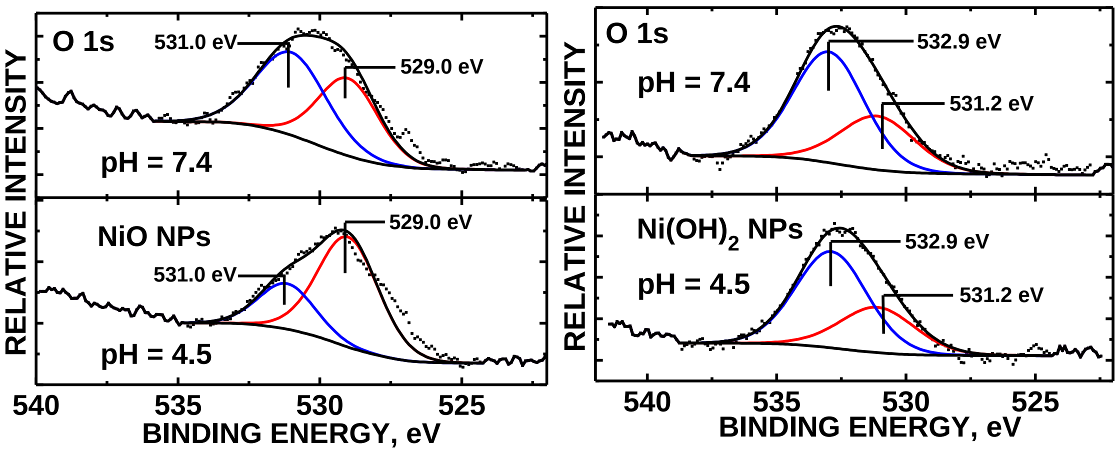

Figure S2. XPS core levels of O 1s orbitals of NiO (left) and $Ni(OH)_2$ (right) nanoparticles.

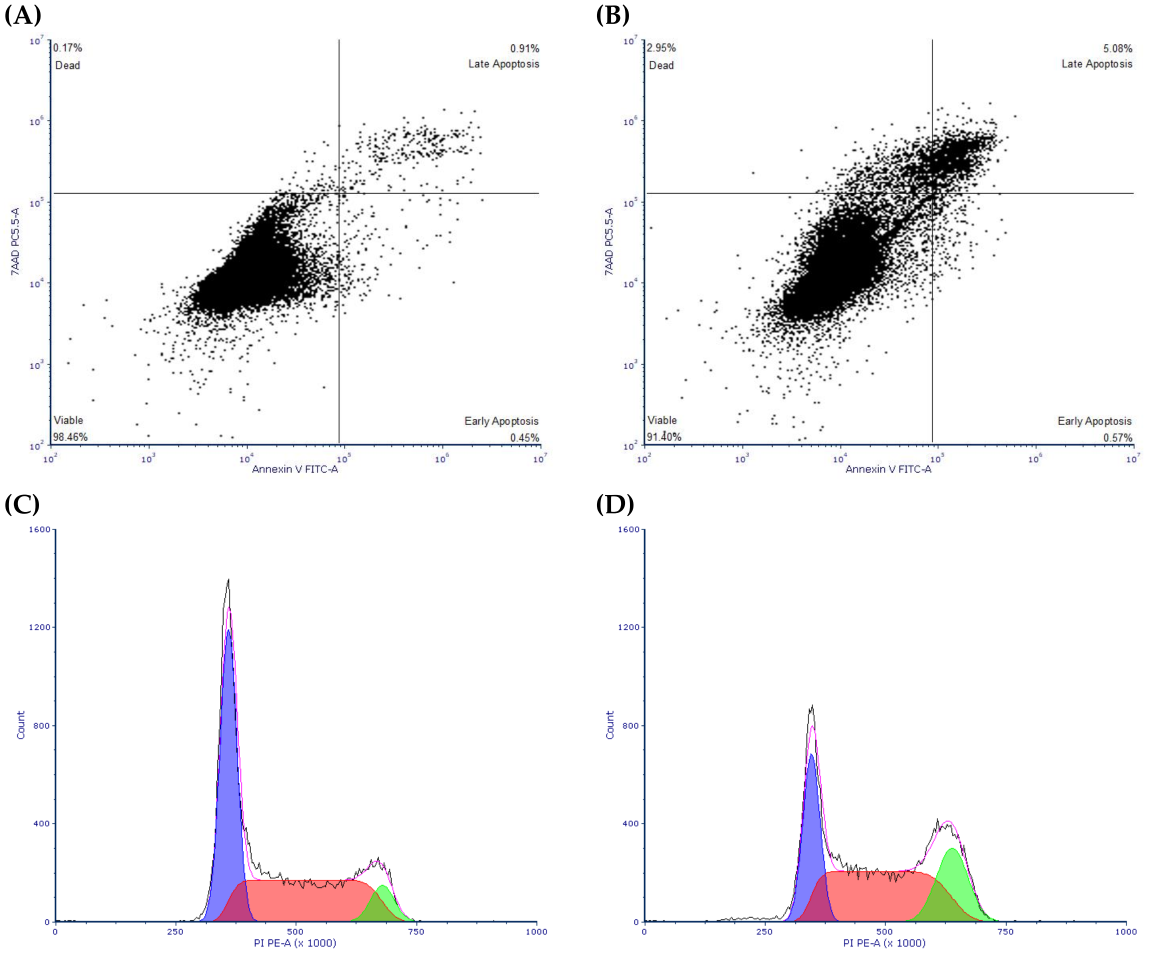

Figure S3. Exemplary flow cytometry data in FCS Express 6. $Ni(OH)_2$ apoptosis analysis after 24h exposure to (**A**) 0 and (**B**) 100 μg/mL. $Ni(OH)_2$ cell cycle analysis after 24h exposure to (**C**) 0 and (**D**) 100 μg/mL.

Table S1. Point of Zero Charge.

Nanoparticle		Initial pH												
		**1.16**	**1.94**	**2.96**	**3.89**	**4.64**	**6.2**	**6.81**	**6.8**	**8**	**9.4**	**10.83**	**11.83**	**12.56**
**NiO**	**Final pH**	1.53	3.47	6.04	8.34	8.59	8.72	8.72	8.74	8.8	8.77	9.75	11.75	12.7
**$Ni(OH)_2$**		1.32	4.86	7.14	7.85	7.89	7.91	7.9	7.94	7.88	7.91	8.28	11.72	12.73

Table S2. HepG2 Viability.

	Nanoparticle Concentration (μg/mL)					
**Nanoparticle**	**0**	**10**	**25**	**50**	**75**	**100**
**NiO 24h**	100.0 ± 0.0	100.3 ± 1.9	103.2 ± 1.3	98.5 ± 0.7	102.2 ± 1.2	99.2 ± 2.5
**$Ni(OH)_2$ 24h**	100.0 ± 0.0	102.2 ± 1.4	103.9 ± 4.4	99.9 ± 2.1	98.9 ± 1.6	96.9 ± 1.6
**NiO 48h**	100.0 ± 0.0	107.3 ± 3.2	105.1 ± 0.9	101.3 ± 1.7	99.5 ± 1.9	93.7 ± 7.1
**$Ni(OH)_2$ 48h**	100.0 ± 0.0	93.8 ± 7.5	86.1 ± 13.1	86.1 ± 12.4	74.6 ± 11.1	72.1 ± 10.6

Data are expressed at the mean ± the SD.
